# Intradermal Inoculation of Inactivated Foot-and-Mouth Disease Vaccine Induced Effective Immune Responses Comparable to Conventional Intramuscular Injection in Pigs

**DOI:** 10.3390/vaccines12020190

**Published:** 2024-02-13

**Authors:** Simin Lee, Sameer ul Salam Mattoo, Chang-Gi Jeong, Seung-Chai Kim, Salik Nazki, Gyehan Lee, Yong-Soo Park, Sun Young Park, Myeon-Sik Yang, Bumseok Kim, Sang-Myeong Lee, Won-Il Kim

**Affiliations:** 1 College of Veterinary Medicine, Jeonbuk National University, 79 Gobong-ro, Iksan 54596, Republic of Korea; lunark321@gmail.com (S.L.); jcg0102@gmail.com (C.-G.J.); leesor2@jbnu.ac.kr (S.-C.K.); saliknazki@gmail.com (S.N.); 2 Division of Biotechnology, Jeonbuk National University, 79 Gobong-ro, Iksan 54596, Republic of Korea; drsameerulsalam@gmail.com; 3 Pandemic Sciences Institute, Nuffield Department of Medicine, University of Oxford, Oxford OX3 7DQ, UK; 4 Miraclescope Inc., 700 Pangyo-ro, Seongnam 13516, Republic of Korea; sky9409@gmail.com; 5 Department of Livestock, Korea National University of Agriculture and Fisheries, Jeonju 54874, Republic of Korea; dvmpys@korea.kr; 6 Animal and Plant Quarantine Agency, 177 Hyeoksin 8-ro, Gimcheon 39660, Republic of Korea; sun3730@korea.kr; 7 Department of Companion and Laboratory Animal Science, Kongju National University, Yesan-eup, Yesan 32439, Republic of Korea; 111@kongju.ac.kr; 8 Biosafety Research Institute and Laboratory of Veterinary Pathology, College of Veterinary Medicine, Jeonbuk National University, 79 Gobong-ro, Iksan 54596, Republic of Korea; bskims@jbnu.ac.kr; 9 College of Veterinary Medicine, Chungbuk National University, 1 Chungdae-ro, Chungju 28644, Republic of Korea

**Keywords:** needle-free injector, intradermal vaccination, foot-and-mouth disease, vaccine, immune response

## Abstract

All pigs in the Republic of Korea are given the foot-and-mouth disease virus (FMDV) vaccine intramuscularly (IM) as part of the country’s vaccination policy. However, the IM administration of the FMDV vaccine to pig results in residual vaccine components in the muscle and undesirable changes in muscle and soft tissues, causing economic losses in swine production. In this study, we evaluated whether intradermal (ID) vaccination could be proposed as an alternative to IM administration. ID vaccination (0.2 mL on each side of the neck muscle) and IM vaccination (2 mL on each side of the neck muscle) were performed twice, separated by 14 days, using a commercial FMD vaccine in specific-pathogen-free pigs. We observed growth performance, gross and microscopic lesions at the inoculation site, FMDV-specific antibodies, and neutralizing antibodies for 35 days after vaccination. Side effects on the skin grossly appeared following ID administration, but most were reduced within two weeks. All ID-vaccinated pigs showed inflammatory lesions limited to the dermis, but IM-vaccinated pigs had abnormal undesirable changes and pus in the muscle. ID-vaccinated pigs performed comparably to IM-vaccinated pigs in terms of growth, FMD virus-specific antibodies, protection capability against FMDV, and T-cell induction. This study demonstrated that the ID inoculation of the inactivated FMD vaccine induced immune responses comparable to an IM injection at 1/10 of the inoculation dose and that the inoculation lesion was limited to the dermis, effectively protecting against the formation of abnormal undesirable changes in muscle and soft tissues.

## 1. Introduction

Foot-and-mouth disease (FMD) is caused by the foot-and-mouth disease virus (FMDV) and is highly contagious in cloven-hooved animal species, including cattle, pigs, goats, and other wild species [1,2]. FMD is characterized by the formation of vesicles on the snout, lips, interdigital clefts of the feet, or on non-haired skin, such as the muzzle and teats [3]. This disease lowers the growth rate, milk production, and mobility of infected animals, affecting livestock productivity [1]. Moreover, the economic cycle is perpetuated by increases in the production cost of meat and corresponding decreases in consumer demand [4].

Massive outbreaks of FMD in the Republic of Korea from 2010 to 2011 led to the implementation of various policies to break this economic cycle; specifically, the Republic of Korea has adopted policies of intramuscularly (IM) vaccinating all susceptible livestock with an inactivated FMD vaccine [5,6]. The adjuvants included in the vaccine regulate the immune response to the vaccine through inducing proinflammatory cytokine production and regulating the T-helper 1 (Th1)/T-helper 2 (Th2) balance, but the immune activation mediated by these adjuvants may lead to various local or systemic reactions, including strong immune response, potentially causing adverse effects [7]. Therefore, excessive immune responses induced by preventive vaccines may lead to undesirable muscle and soft tissue changes in livestock that reduce meat quality, including granuloma, sterile abscess, residual vaccine, and nodular lesions in the muscle layer [6,8]. Undesirable vaccination-induced muscle and soft tissue changes persist for a long time and are observed even in slaughterhouses. In accordance with the detailed inspection standards of the Livestock Products Sanitary Control Act, defective parts of muscle and soft tissues are discarded [9]. The resulting economic damage is estimated to amount to USD 115 million per year [10]. 

Needle-free intradermal (ID) vaccination has been used as an alternative to the traditional needle and syringe method. In contrast to IM vaccination, ID vaccination is administered subcutaneously rather than intramuscularly, reducing the changes in muscle and soft tissues and preventing economic losses in meat production [11]. Moreover, needle-free ID vaccination is less painful, avoids lesions in the muscle and reduces vaccination time as needle replacement is not required [6]. Given these advantages, the efficacy of the ID administration of the FMDV vaccine using a needle-free ID injector prototype was evaluated in this study based on side effects, body weight gain, and the induction of antibodies and T-cell immune responses.

## 2. Materials and Methods

### 2.1. Intradermal Jet Injector and Vaccine Information

The needle-free ID injector (Mirajet-100, Miraclescope, Seongnam, Republic of Korea) used in the present study was developed for needle-free injection. This injector is driven by a brushless direct current motor, and vaccine vials of various sizes (20, 50, and 100 mL) can be directly loaded to the injector. This injector dispenses vaccines at pressures exceeding 11 MPa and allows for adjustable operation speeds, facilitating the use of oil adjuvants commonly employed with FMDV vaccines. To increase the portability of the injector, a removable battery was introduced, and the injector was manufactured with a weight of less than 1700 g and a size of 290 × 260 mm (Figure S1).

The commercial FMDV vaccine (Aftogen^®^ Oleo) used in the vaccine program of the Republic of Korea was used in the current study. This monovalent FMD vaccine was manufactured by Biogénesis Bagó in Argentina and contains inactivated FMDV O1 Campos antigens in an oil-in-water emulsion (>6 protective dose 50 [PD_50_]). The O1 Campos vaccine strain is able to efficiently cross-protect against three topotypes of FMDV currently circulating in Asia (SEA, ME-SA, and CATHAY), including field isolates from 2010 and 2014⁠–2015 in the Republic of Korea [12]. Based on these results, Aftogen^®^ Oleo has been used in vaccine programs in pigs in the Republic of Korea since 2016.

### 2.2. Animals and Experimental Design

Six 19-week-old specific-pathogen-free (SPF) pigs (FMDV-free, porcine circovirus 2-free, *Mycoplasma hyopneumoniae*-free, and porcine reproductive and respiratory syndrome virus-free) were purchased from Optifarm (Osong, Republic of Korea). Pigs were divided into two groups of 3 pigs: the ID group and IM group. After three days of acclimatization, the ID group received 0.2 mL of a vaccine on each side of the neck muscle through the ID route, while the IM group received 2 mL of a vaccine on each side of the neck muscle through the IM route. The abovementioned needle-free intradermal injector and a syringe were used for ID and IM vaccination, respectively. The groups were housed in separate rooms with unlimited water and feed during the study period. At 14 days post-vaccination (DPV), all pigs were given a booster dose of vaccine equal to the primary vaccination. Blood samples were collected from the pigs, and they were monitored at regular intervals after vaccination. Pigs were slaughtered humanely after sedation by azaperone (intramuscular injection, 1 mL/20 kg) at 35 DPV. This animal experiment was approved by the Jeonbuk National University Institutional Animal Care and Use Committee (Approval Number: JBNU 2021-095).

### 2.3. General Observation and Sample Preparation

The general health of all animals was monitored daily up to 35 DPV. All pigs were weighed, and the average daily weight gain (ADWG) was evaluated each week in the same manner as in the previous experiment [11]. Blood samples were collected at 0, 7, 14, 21, 28, and 35 DPV. Injection sites were evaluated for local adverse effects, such as redness, the formation of small swellings, and other gross findings on 1 (the day after primary immunization), 15 (the day after secondary immunization), and 35 (the day of necropsy) DPV. On the necropsy day, the injection site was dissected to assess the presence of undesirable changes in muscle and soft tissues, and specimens were collected for histological examination. The local injection site reaction (ISR) score was evaluated on the day of necropsy based on a previous study with some modifications [13]. Briefly, the previous study evaluated six categories (i.e., redness, the size of redness, swelling, pain during palpation, necrosis/ulcer, and induration) as side effects. We added a new category, abscess/pus in the injection site, which was scored from 0 to 2 based on severity.

### 2.4. Histopathology

Skin and muscle, including the injection site, were harvested and fixed in a 5% formalin solution. Tissues were embedded in paraffin wax (Surgipath Paraplast, Leica Biosystems) and cut to a thickness of 4 μm. The tissue slices were transferred onto a glass slide, deparaffinized using xylene and ethanol washes, and rehydrated. Finally, these slides were stained with hematoxylin and eosin (HE). A confocal microscope (LSM 900, Carl Zeiss) was used to examine the stained tissue and capture images at 100× or 200× magnification.

### 2.5. ELISA and Virus Neutralizing Test

Anti-FMDV antibodies against serotype O in serum samples were evaluated using a commercially available ELISA kit (PrioCHECK™ FMDV type O Antibody ELISA kit; ThermoFisher Scientific) according to the manufacturer’s instructions. The percentage inhibition (PI) of the tested serum and reference serum was calculated from the measured optical density at 450 nm. Samples with a PI value ≥ 50% were considered to be positive for the FMDV antibody.

The virus-neutralizing test proceeded in the same manner as in the previous paper [6]. Briefly, serum inactivated at 56 °C for 30 min was serially diluted in a 96-well cell culture plate and mixed with 100 TCID_50_ of an O1 Manisa virus suspension. The prepared mixture was incubated for 1 h, then LF-BK cells were added and incubated for an additional 3 days. Virus-neutralizing (VN) antibody titers were measured based on the cytopathic effect of LF-BK cells, and sera with a titer of more than 1.2 log_10_ were considered antibody-positive.

### 2.6. Animal Experiment with Conventional Pigs

To validate the field application of the vaccination method, an additional experiment was conducted on six 4-week-old conventional pigs. Except for the omission of virus-neutralization testing and flow cytometric analysis, every step was performed consistently with the SPF pig experiments. Since the purpose of this experiment was to compare the efficacy of ID and IM preventive vaccinations, the results of the SPF pig experiment were highlighted in the main text without comparing the experimental outcomes of conventional pigs. The additional experimental results with conventional pigs are provided in the Supplementary Materials, and a detailed discussion of these findings is presented in the Section 4.

### 2.7. Data Analysis and Statistical Methods

We hypothesized that the ID group would have lower outcomes than the IM group as our null hypothesis and posited as our alternative hypothesis that there would be no difference between the results of the ID and IM groups. To compare the ADWG at 35 DPV between the ID and IM groups, we employed a non-parametric t-test equivalent (i.e., the Mann–Whitney U test). Weight and serology were evaluated using a two-way analysis of variance (ANOVA) to assess differences according to the vaccination method and DPV. Within the framework of the two-way ANOVA, comparisons were made between columns (i.e., the group means) and within each row (i.e., different time points). As only two groups were compared, no post hoc tests were conducted. All data are presented as the mean ± standard error of the mean (SEM) in figures and tables. Differences were considered statistically significant at *p* < 0.05. GraphPad Prism 7.00 (GraphPad, San Diego, CA, USA) was utilized for the graphical representation and statistical analysis of the data.

## 3. Results

### 3.1. General Observation

**Figure 1:**
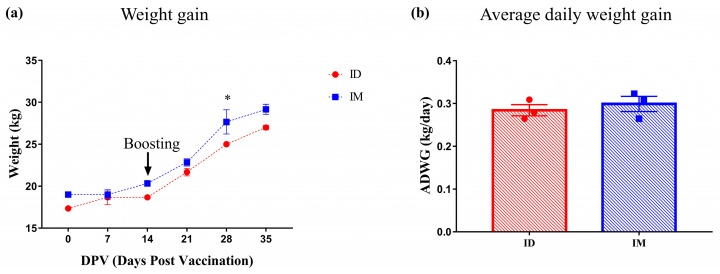
Changes in SPF pig weight and average daily weight gain on the day of necropsy (35 DPV) for each group. (**a**) Weight of SPF pigs; (**b**) ADWG on the necropsy day (35 DPV). Statistical significance between ID and IM groups is indicated by asterisk(s) (* *p* < 0.05).

Pigs were monitored daily for adverse events after immunization. No unusual clinical signs or symptoms, such as anaphylaxis, change in appetite, abnormal breathing, or pain-related behaviors, were seen in any pigs during the study period. The SPF pigs continued to gain weight during the experiment, and at 28 DPV, the weight of the IM group was significantly higher than that of the ID group, but significance was not observed afterward (Figure 1a). There was no significant difference in ADWG between the ID (0.28 ± 0.01 kg/day) and IM (0.30 ± 0.02 kg/day) groups (Figure 1b). 

**Figure 2:**
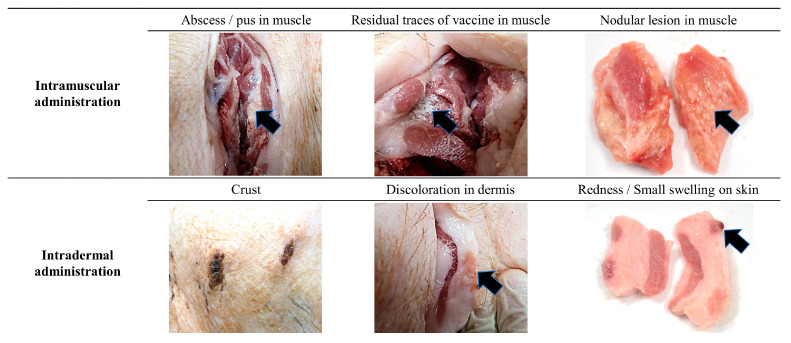
Typical gross lesions observed at the intramuscular and intradermal vaccination site. Compilation of representative images captured during necropsy (on 35 DPV), illustrating typical observations at the vaccination site.

Local reactions were not observed at the primary vaccination site on the skin of any of the SPF pigs in the IM group throughout the study period (Figure S2). On the day following secondary immunization (15 DPV), redness with a diameter of less than 1 cm was observed on the skin of one SPF pig in the IM group at the secondary vaccination site (1/3; 33.3%). This observed redness subsided and was no longer present on the day of necropsy (0/3; 0%); however, discoloration on the skin less than 1 cm in diameter was observed at the primary and secondary vaccination site (3/3; 100%). When the vaccination site was dissected, abscesses and nodular lesions were observed in the muscle layer of all pigs in the IM group (3/3; 100%), and residual traces of vaccine were observed in the muscle layer of two pigs in the IM group (2/3; 66.7%) (Figure 2). 

After the primary vaccination in the ID group, the primary vaccination site exhibited crusts (2/3; 66.7%), redness (2/3; 66.7%), and small swellings (2/3; 66.7%) on the following day (Figure S2). Redness and small swellings at this site subsided in all pigs after 14 DPV. However, the crust persisted until the necropsy day in two SPF pigs from the ID group. At the secondary vaccination site, crusts (2/3; 66.7%), redness (3/3; 100%), and small swellings (1/3; 33.3%) were observed on the day after the second vaccination (15 DPV). Redness was not observed in any pigs (0/3; 0%), while crusts (3/3; 100%) and small swellings (1/3; 33.3%) persisted until the day of necropsy. Upon the dissection of the vaccination site, discoloration was observed in the dermis of two pigs in the ID group (2/3; 66.7%), but no abnormal findings were noted in the muscle layer (Figure 2).

### 3.2. Local Injection Site Reaction (ISR) Score

The ISR score at the day of necropsy was higher in the IM group than in the ID group, notwithstanding gross findings on the skin during the experimental period (Table 1). One pig in the IM group had slight redness of less than 1 cm in diameter (score 1) at necropsy. At the vaccination site in the IM group, there was neither swelling (score 0) nor necrosis/ulcers (score 0). Two pigs in the IM group showed slight pain (score 1) when the area was palpated, and we detected firmness (score 1). Pus and abscesses (score 2) were found in the muscle of all pigs in the IM group. In contrast, redness, pain, necrosis/ulcer, and abscess/pus were not observed in any ID pigs except for slight and firm swelling (score 1) on the skin of one pig.

### 3.3. Histopathology

**Figure 3:**
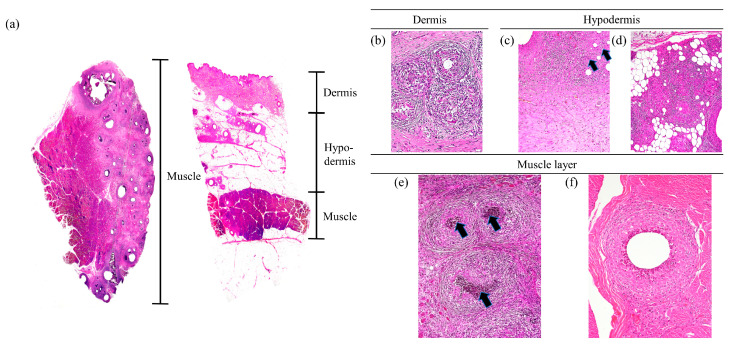
Representative histopathology of side effects at the vaccination site of intramuscular injection. (**a**) Image of stained tissue. Side effects were mainly observed in the hypodermis and muscle layer. (**b**) Granulomas in the dermis; 200× magnification. (**c**) Proliferated collagen, lymphocytes, macrophages, and multinucleated giant cells (arrows) were observed in the hypodermis; 100× magnification. (**d**) Granulomas in the hypodermis. Collagen, pink extracellular matrix, and inflammatory cells are spread out the center of granuloma and adipose tissue; 100× magnification. (**e**) Pyogranulomas in the muscle layer. Central necrosis is observed in the center of pyogranulomas (arrows); 100× magnification. (**f**) Adjuvant remaining in the muscle; 100× magnification.

**Figure 4:**
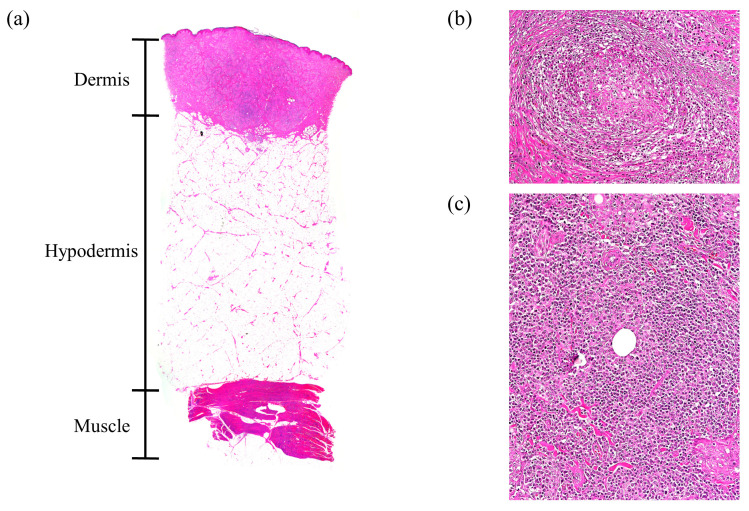
Representative histopathology of side effects at the vaccination site of the intradermal injection. (**a**) Stained tissue image. Side effects were limited to the dermis. (**b**) Granuloma in the dermis. Collagen, pink extracellular matrix, and inflammatory cells, including lymphocytes, neutrophils, and macrophages were induced in the dermis. Fibroblasts formed thin connective tissue walls that surrounded and isolated immune cells; 100× magnification. (**c**) Granulomatous inflammation in the dermis, lymphocytes, neutrophils, and macrophages induced by the inflammatory reaction were randomly distributed around residual traces of adjuvant; 100× magnification.

The predominant histopathological observations at the vaccination site were inflammatory reactions and various sizes of inflammatory foci (granuloma) (Figure 3 and Figure 4). Histopathological lesions in the IM group were not limited to the muscle layer and were also observed in the hypodermis (2/3; 66.7%) and dermis (1/3; 33.3%). Pyogranulomas, as well as granulomas, were observed in two pigs of the IM group (2/3; 66.7%). In addition, residual traces of the vaccine were distributed from the muscle layer to the hypodermis (3/3; 100%) in the hollow form surrounded by immune cells. In one pig in the IM group, excessive collagen formation was observed in the hypodermis (Figure 3). Together, the spaces within muscle and adipose tissue formed by granulomas, pyogranulomas, and residual vaccine adjuvant resulted in tissue deformation (Figure 3). However, in all pigs in the ID group, inflammatory reactions and abnormalities were observed only in the dermis, and side effects were not observed in the hypodermis or muscle layer (Figure 4). Boundaries of these granulomas were clear, and the infiltration of inflammatory cells was not observed outside the boundary. In some pigs in the ID group, residual traces of vaccine (1/3; 33.3%) and granulomatous inflammation between granulomas (1/3; 33.3%) were observed in the dermis (Figure 4).

### 3.4. FMDV-Specific Antibody and Neutralizing Antibody Induction

**Figure 5:**
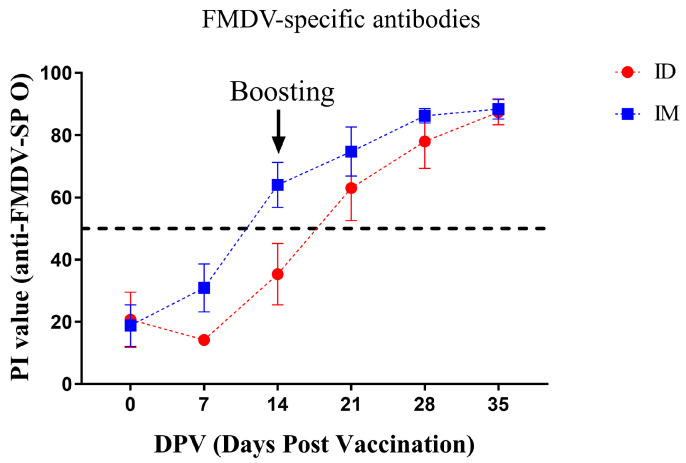
Changes in FMDV-specific antibodies. The % PI value was quantified by a commercial ELISA kit at specific time points.

All pigs in the IM and ID groups were seronegative before primary vaccination, and their PI values were 18.8 ± 6.7% and 20.7 ± 8.9% at 0 DPV, respectively (Figure 5). The PI value of the IM group steadily increased after vaccination, with all pigs being positive at a value of 64.0 ± 7.2% at 14 DPV; the PI value continued to increase thereafter, reaching the highest values (87.4 ± 4.0%) at 35 DPV. The PI value of the ID group underwent a transient decrease at 7 DPV but resumed an upward trend, reaching a value of 63.0 ± 10.5% at 21 DPV, with all pigs subsequently seroconverting. The PI values of the ID group continued to increase, yielding the highest values (88.4 ± 3.2%) at 35 DPV. Significant differences in % PI values were not observed between the ID and IM groups during the experimental period.

**Figure 6:**
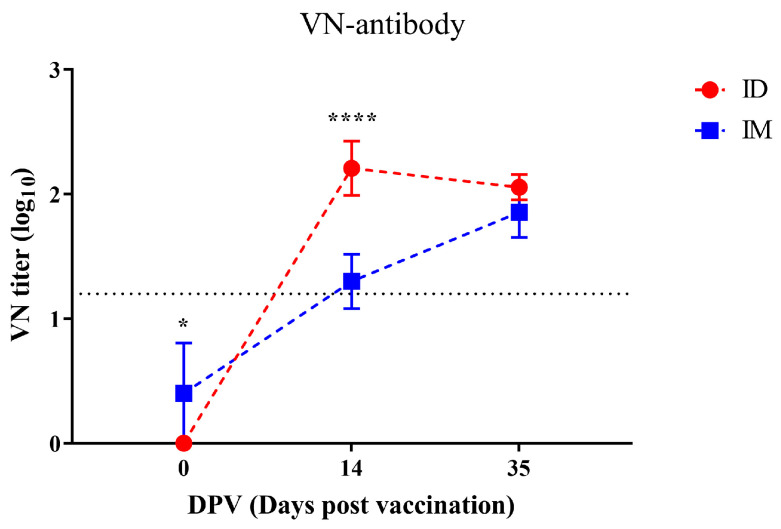
Changes in titers of VN antibody against O1 Manisa. VN titers over 1.2 log_10_ (dotted line) were considered to be protected against FMDV. Statistical significance between ID and IM groups is indicated by asterisk(s) (* *p* < 0.05, **** *p* < 0.0001).

All pigs in the IM and ID groups had negligible titers of VN antibodies before primary vaccination (Figure 6). VN titers of the IM group increased steadily after vaccination, and the average titers above 1.2 log_10_ were observed at 14 DPV except for one pig (1.3 ± 0.2 log_10_). Thereafter, the titer continued to increase, reaching the highest value (1.9 ± 0.2 log_10_) at 35 DPV. VN titers of the ID group increased dramatically after vaccination compared to the IM group, and titers above 1.2 log_10_ were observed in all pigs at 14 DPV, which was significantly higher than that of the IM group (2.2 ± 0.2 log_10_). Thereafter, the titer decreased slightly at 35DPV (2.1 ± 0.1 log_10_).

## 4. Discussion

The expensive equipment costs for ID administration and government regulations have prevented the widespread use of ID immunization on farms in the Republic of Korea. To date, there has been considerable research conducted on this approach for FMDV ID vaccination and the development of a needle-free device [6,14,15]. Cattle that had been ID-vaccinated at a 1/16 dose with a commercially available vaccine formulation showed effective protective ability against the challenge of FMDV [16]. The previous study evaluated test vaccines in pigs composed of various formulations and provided information on adjuvant and formulation selections for the FMDV vaccine [6]. Nevertheless, pigs have been evaluated only for the test vaccine hence the options for ID vaccination of the FMDV vaccine in the swine industry are still limited in the Republic of Korea. Therefore, it was evaluated in this study whether the combination of an easily obtained FMDV commercial vaccine and the needle-free ID injector could replace the conventional FMD vaccination approach used in the Republic of Korea. Additionally, the prospect of increasing the productivity of pigs using a novel vaccination strategy was considered. In this experiment, SPF pigs and conventional pigs were immunized through ID or IM administration to assess productivity, side effects, and changes in the percentage of antibodies and immune cells.

All SPF pigs gained body weight steadily after vaccination, and the ADWG of the ID group was similar to that of the IM group (Figure 1). In a previous study using the same vaccine, differences in ADWG were not observed between the IM-vaccinated and non-vaccinated groups until six weeks after vaccination [17]. The results of this and previous studies confirm that pig productivity does not change with a vaccination regimen. Local adverse reactions induced by vaccination varied depending on the administration route. Redness was noticed on the skin following vaccination in the IM group, while pyogranulomas, abscesses, nodular lesions, and residual traces of vaccine were found in the muscle layer, reducing meat quality (Figure 2 and Figure 3, Table 1). These side effects are mainly due to the substances used as adjuvants [8,18]. Due to the adjuvant, acute inflammation can be induced, leading to the formation of abscesses [19]. The observed abscesses and granulomas mixed with monocytes, macrophages, neutrophils, and eosinophils in the muscle layer of the IM group (Figure 2 and Figure 3) that resulted from the prolonged recovery period, during which the formed abscesses were not removed and persisted [18,19]. If the IM injection were administered in a 1/10 dose similar to the ID group, it is possible that the side effects observed in the muscles might be significantly reduced; however, this would also lead to a decrease in the titer of neutralizing antibodies, making it difficult to achieve sufficient defensive capability [20]. On the other hand, ID vaccination has a lower amount of adjuvant to be administered than IM vaccination; hence, the side effects are limited to the dermis and are milder. Local adverse reactions on the skin are more common after ID immunization than IM immunization [6,21]. Although wounds and crusts were present on the skin at the injection site following ID administration, they were thought to be due to the high pressure of the injected vaccine rather than the vaccine adjuvant, and most of these side effects disappeared within two weeks (Figures S2 and S3, Table 1). Granulomas or abscesses were not observed in the muscles of the ID group, and encapsulated granulomas were observed only in the dermis under a microscope (Figure 2 and Figure 4). Therefore, ID administration using the needle-free injector is excellent in terms of pig welfare and economic feasibility because it reduces the occurrence of undesirable changes in muscle and soft tissues.

The immunogenicity of a vaccine is mainly evaluated with a specific antibody against structural proteins constituting the FMDV capsid and a VN antibody. Antibodies for structural proteins are used as indicators of previous infection or the immunization of the specific FMDV serotype [22]. IgG, the major type of antibody, is induced from 4 to 7 DPV and reaches its maximum level at 21 DPV [23]. Correspondingly, FMDV-specific antibody levels in SPF pigs gradually increased after 7 DPV, and all pigs had seroconverted by 21 DPV, which is consistent with the results of the previous study (Figure 5) [24]. In this experiment, the induction of FMDV-specific antibodies in the ID group lagged behind that in the IM group by approximately one week, suggesting that the priming time of the vaccine in the body differs depending on the administration route [25]; however, there was no difference in FMDV-specific antibody levels between the IM and ID groups at the end of the experiment. Early in FMDV infection, the humoral immune response, including VN antibodies, neutralizes the virus via antibody-dependent mechanisms and induces antibody-dependent cellular cytotoxicity through opsonization [26,27]. Therefore, VN antibodies are used as a criterion for the formation of protective capabilities against the corresponding FMDV serotype. Since the antigen of O1 Campos was not available in this experiment, VN antibody titers against O1 Manisa, which has high cross-reactivity, were measured instead [28]. Surprisingly, the induction of VN antibodies in the ID group was initially higher than that in the IM group and maintained at a comparable level until the end of the experiment (Figure 6). Accordingly, the ID group was evaluated to be immunogenic against homologous antigens. However, the fact that the induction of VN antibodies can be improved by controlling the type and amount of adjuvant indicates the need for further in-depth research on the development of an ID vaccine for better VN antibody induction than this experiment [29,30]. Comprehensively, ID administration using the ID injector achieved vaccine priming as effective as IM vaccination and rapidly induced high VN antibody titers to protect the host at an early stage of FMDV infection, even at 1/10 of the dose. Nevertheless, further studies are needed to evaluate the appropriate doses and formulations of antigens and adjuvants to induce effective protection against FMDV.

In addition to the comparative evaluations of ID and IM vaccination in SPF pigs, a similar experiment was performed further with 4-week-old conventional pigs. This additional study aimed to demonstrate that ID vaccination could be as effective as IM vaccination in the field while causing fewer and non-severe adverse effects. All conventional pigs used in the additional study had maternally derived antibodies because of the Republic of Korea’s FMD vaccine policy. After ID or IM vaccination, the pigs continued to gain weight until the end of the experiment without any significant difference between the ID (0.67 ± 0.03 kg/day) and IM (0.53 ± 0.08 kg/day) groups after vaccination (Figure S4). During the study period, redness or skin discoloration (2/3; 66.6%) were observed in the IM group at the vaccinated site, but abscesses and nodular lesions (3/3; 100%) were located in the muscle layer at the incised vaccinated site (Figure S3). Conversely, abnormal findings were not observed in the muscle layer of all pigs in the ID group except for crusts, redness, small swellings, and dermal discoloration on the skin (Figure S3). Even microscopically, the inflammatory response and granulomas were confined to the muscle layer and the dermis in the IM and ID groups, respectively (data not shown). All conventional pigs were seropositive before the primary vaccination (Figure S4). The % PI values in the IM group stayed constant for two weeks post-vaccination, then steadily increased to 92.5 ± 2.0% until 35 DPV. The % PI value of the ID group temporarily decreased after vaccination but increased sharply from 21 DPV, recording a peak of 91.8 ± 3.3% at 35 DPV. Overall, the ID group had superior production and no undesirable changes in muscle and soft tissues compared to the IM group. The FMD-specific antibodies in both the ID and IM groups increased in a similar trend, replacing the gradually decreasing maternal-derived antibodies.

In this study, we endeavored to minimize the number of animals used, adhering to less stringent assumptions about error levels and employing the non-parametric Mann–Whitney U test, in alignment with animal welfare principles and ethical research practices. [31]. This deliberate limitation, while ensuring humane treatment of animals, could potentially affect the robustness and generalizability of our findings. The small sample size may limit the study’s statistical power and potentially impact the detection of significant differences between groups [31]. This aspect emphasizes the need for careful consideration when extrapolating these results to a broader context.

In conclusion, the intradermal inoculation of the inactivated FMD vaccine using the needle-free ID injector increased productivity and effectively induced protection and immune cell activation against FMDV without the undesirable changes in muscle and soft tissues, in contrast to IM administration. Furthermore, the ID administration of commercial FMDV vaccines using the developed ID injector is expected to facilitate a high FMDV vaccination rate in pigs through rapid and accurate vaccination because it is more accessible and easier to use than conventional IM administration. While the limited sample size of this study calls for careful interpretation of the results, the superiority of ID administration demonstrated in this study could play an important role in facilitating the widespread use of ID vaccination in the future.

## Figures and Tables

**Table 1:** Local injection site reaction score in SPF pigs at 35 DPV.

Group	No.	Redness		Size of Redness		Swelling		Pain during Palpation		Necrosis/Ulcer		Induration		Abscess/Pus	
		Score	Mean ± SEM	Score	Mean ± SEM	Score	Mean ± SEM	Score	Mean ± SEM	Score	Mean ± SEM	Score	Mean ± SEM	Score	Mean ± SEM
IM	1	0	0.33 ± 0.33	0	0.33 ± 0.33	0	0	1	0.67 ± 0.33	0	0	1	0.67 ± 0.33	2	2 ± 0
	2	0		0		0		0		0		0		2	
	3	1		1		0		1		0		1		2	
ID	1	0	0	0	0	1	0.33 ± 0.33	0	0	0	0	1	0.33 ± 0.33	0	0
	2	0		0		0		0		0		0		0	
	3	0		0		0		0		0		0		0	

## Data Availability

Data available within the article or its Supplementary Materials.

## Supplementary Materials

The following supporting information can be downloaded at: https://www.mdpi.com/article/10.3390/vaccines12020190/s1, Figure S1: Needle-free ID injector developed and evaluated in the current study; Figure S2: Local reactions at the vaccination site in the ID- and IM-vaccinated pigs during the experiment (SPF pigs); Figure S3: Local reactions at the vaccination site in the ID- and IM-vaccinated pigs during the experiment (conventional pigs); Figure S4: The changes in weight gain and FMDV-specific antibodies after immunization of the ID- and IM-vaccinated groups in conventional pigs.

## References

[1] Diaz-San Segundo F., Medina G.N., Stenfeldt C., Arzt J., de Los Santos T. (2017). Foot-and-Mouth Disease Vaccines. Vet. Microbiol..

[2] Li P., Bai X., Sun P., Li D., Lu Z., Cao Y., Fu Y., Bao H., Chen Y., Xie B. (2012). Evaluation of a Genetically Modified Foot-and-Mouth Disease Virus Vaccine Candidate Generated by Reverse Genetics. BMC Vet. Res..

[3] Arzt J., Juleff N., Zhang Z., Rodriguez L.L. (2011). The Pathogenesis of Foot-and-Mouth Disease I: Viral Pathways in Cattle. Transbound. Emerg. Dis..

[4] Gim U.-S., Choi S.-H., Cho J.-H. (2015). An Impact Analysis of FMD News on Pork Demand in Korea. Korean J. Community Living Sci..

[5] Park J.-H., Lee K.-N., Ko Y.-J., Kim S.-M., Lee H.-S., Shin Y.-K., Sohn H.-J., Park J.-Y., Yeh J.-Y., Lee Y.-H. (2013). Control of Foot-and-Mouth Disease during 2010-2011 Epidemic, South Korea. Emerg. Infect. Dis..

[6] Hwang J.-H., Lee K.-N., Kim S.-M., Lee G., Moon Y., Kim B., Lee J.-S., Park J.-H. (2019). Needleless Intradermal Vaccination for Foot-and-Mouth Disease Induced Granuloma-Free Effective Protection in Pigs. J. Vet. Sci..

[7] Batista-Duharte A., Portuondo D., Carlos I.Z., Pérez O. (2013). An Approach to Local Immunotoxicity Induced by Adjuvanted Vaccines. Int. Immunopharmacol..

[8] Ko E.Y., Jung S., Jeong H.K., Han J.H., Son J.H. (2018). Effects of Foot-and-Mouth Disease Vaccination Location and Injection Device on the Incidence of Site Lesions in Pork. Korean J. Food Sci. Anim. Resour..

[9] Animal and Plant Quarantine Agency (2016). Field Study of FMD Vaccination Using Needless Transdermal Device.

[10] Choi S.-H., Pak S.-I. (2015). Economic Burden of Foot-and-Mouth Disease Vaccination-Induced Injection Site Lesions in Slaughtered Pigs and Its Causal Relationship. J. Prev. Vet. Med..

[11] Lee S.-I., Jeong C.-G., Ul Salam Mattoo S., Nazki S., Prasad Aganja R., Kim S.-C., Khatun A., Oh Y., Noh S.-H., Lee S.-M. (2021). Protective Immunity Induced by Concurrent Intradermal Injection of Porcine Circovirus Type 2 and Mycoplasma Hyopneumoniae Inactivated Vaccines in Pigs. Vaccine.

[12] Galdo Novo S., Malirat V., Maradei E.D., Pedemonte A.R., Espinoza A.M., Smitsaart E., Lee K.N., Park J.H., Bergmann I.E. (2018). Efficacy of a High Quality O1/Campos Foot-and-Mouth Disease Vaccine upon Challenge with a Heterologous Korean O Mya98 Lineage Virus in Pigs. Vaccine.

[13] Stadler J., Naderer L., Beffort L., Ritzmann M., Emrich D., Hermanns W., Fiebig K., Saalmüller A., Gerner W., Glatthaar-Saalmüller B. (2018). Safety and Immune Responses after Intradermal Application of Porcilis PRRS in Either the Neck or the Perianal Region. PLoS ONE.

[14] Madapong A., Saeng-Chuto K., Chaikhumwang P., Tantituvanont A., Saardrak K., Pedrazuela Sanz R., Miranda Alvarez J., Nilubol D. (2020). Immune Response and Protective Efficacy of Intramuscular and Intradermal Vaccination with Porcine Reproductive and Respiratory Syndrome Virus 1 (PRRSV-1) Modified Live Vaccine against Highly Pathogenic PRRSV-2 (HP-PRRSV-2) Challenge, Either Alone or in Combination with of PRRSV-1. Vet. Microbiol..

[15] Temple D., Escribano D., Jiménez M., Mainau E., Cerón J.J., Manteca X. (2017). Effect of the Needle-Free “Intra Dermal Application of Liquids” Vaccination on the Welfare of Pregnant Sows. Porc. Health Manag..

[16] Pandya M., Pacheco J.M., Bishop E., Kenney M., Milward F., Doel T., Golde W.T. (2012). An Alternate Delivery System Improves Vaccine Performance against Foot-and-Mouth Disease Virus (FMDV). Vaccine.

[17] Upadhaya S.D., Kim Y.M., Shi H., Le Cour Grandmaison J., Blanchard A., Kim I.H. (2020). Standardized Plant Extract Alleviates the Negative Effects of FMD Vaccination on Animal Performance. Animals.

[18] Valtulini S., Macchi C., Ballanti P., Cherel Y., Laval A., Theaker J.M., Bak M., Ferretti E., Morvan H. (2005). Aluminium Hydroxide-Induced Granulomas in Pigs. Vaccine.

[19] Ackermann M.R., Zachary J.F. (2017). Inflammation and Healing. Pathologic Basis of Veterinary Disease.

[20] Eblé P.L., Weerdmeester K., van Hemert-Kluitenberg F., Dekker A. (2009). Intradermal vaccination of pigs against FMD with 1/10 dose results in comparable vaccine efficacy as intramuscular vaccination with a full dose. Vaccine.

[21] Schnyder J.L., De Pijper C.A., Garcia Garrido H.M., Daams J.G., Goorhuis A., Stijnis C., Schaumburg F., Grobusch M.P. (2020). Fractional Dose of Intradermal Compared to Intramuscular and Subcutaneous Vaccination—A Systematic Review and Meta-Analysis. Travel Med. Infect. Dis..

[22] Ludi A.B., Morris A., Gubbins S., Asfor A., Afzal M., Browning C.F., Grazioli S., Foglia E.A., Wilsden G., Burman A. (2022). Cross-Serotype Reactivity of ELISAs Used to Detect Antibodies to the Structural Proteins of Foot-and-Mouth Disease Virus. Viruses.

[23] Lee M.J., Jo H., Park S.H., Ko M.-K., Kim S.-M., Kim B., Park J.-H. (2020). Advanced Foot-And-Mouth Disease Vaccine Platform for Stimulation of Simultaneous Cellular and Humoral Immune Responses. Vaccines.

[24] Park M.-E., You S.-H., Lee S.-Y., Lee K.-N., Ko M.-K., Choi J.-H., Kim B., Lee J.-S., Park J.-H. (2017). Immune Responses in Pigs and Cattle Vaccinated with Half-Volume Foot-and-Mouth Disease Vaccine. J. Vet. Sci..

[25] Combadiere B., Liard C. (2011). Transcutaneous and Intradermal Vaccination. Hum. Vaccin..

[26] Rodríguez-Habibe I., Celis-Giraldo C., Patarroyo M.E., Avendaño C., Patarroyo M.A. (2020). A Comprehensive Review of the Immunological Response against Foot-and-Mouth Disease Virus Infection and Its Evasion Mechanisms. Vaccines.

[27] Tewari A., Jain B. (2019). Antiviral Immunity Evoked Post Foot-and-Mouth Disease Virus (FMDV) Infection and Vaccination. J. Antivir. Antiretrovir..

[28] Nagendrakumar S.B., Srinivasan V.A., Madhanmohan M., Yuvaraj S., Parida S., Di Nardo A., Horsington J., Paton D.J. (2011). Evaluation of Cross-Protection between O1 Manisa and O1 Campos in Cattle Vaccinated with Foot-and-Mouth Disease Virus Vaccine Incorporating Different Payloads of Inactivated O1 Manisa Antigen. Vaccine.

[29] Choi J.-H., You S.-H., Ko M.-K., Jo H.E., Shin S.H., Jo H., Lee M.J., Kim S.-M., Kim B., Lee J.-S. (2020). Improved Immune Responses and Safety of Foot-and-Mouth Disease Vaccine Containing Immunostimulating Components in Pigs. J. Vet. Sci..

[30] Khorasani A., Madadgar O., Soleimanjahi H., Keyvanfar H., Mahravani H. (2016). Evaluation of the Efficacy of a New Oil-Based Adjuvant ISA 61 VG FMD Vaccine as a Potential Vaccine for Cattle. Iran. J. Vet. Res..

[31] Richter V., Muche R., Mayer B. (2018). How much confidence do we need in animal experiments? Statistical assumptions in sample size estimation. J. Appl. Anim. Welf Sci..

