# Supplementary material for: Intradermal Inoculation of Inactivated Foot-and-Mouth Disease Vaccine Induced Effective Immune Responses Comparable to Conventional Intramuscular Injection in Pigs
Source: Vaccines (Basel). 2024 Feb 13;12(2):190. doi: 10.3390/vaccines12020190 (PMC10892606; doi:10.3390/vaccines12020190)
Supplement: Supplementary file 1 [file vaccines-12-00190-s001.zip › vaccines-2789294-supplementary Figure S3.pdf]

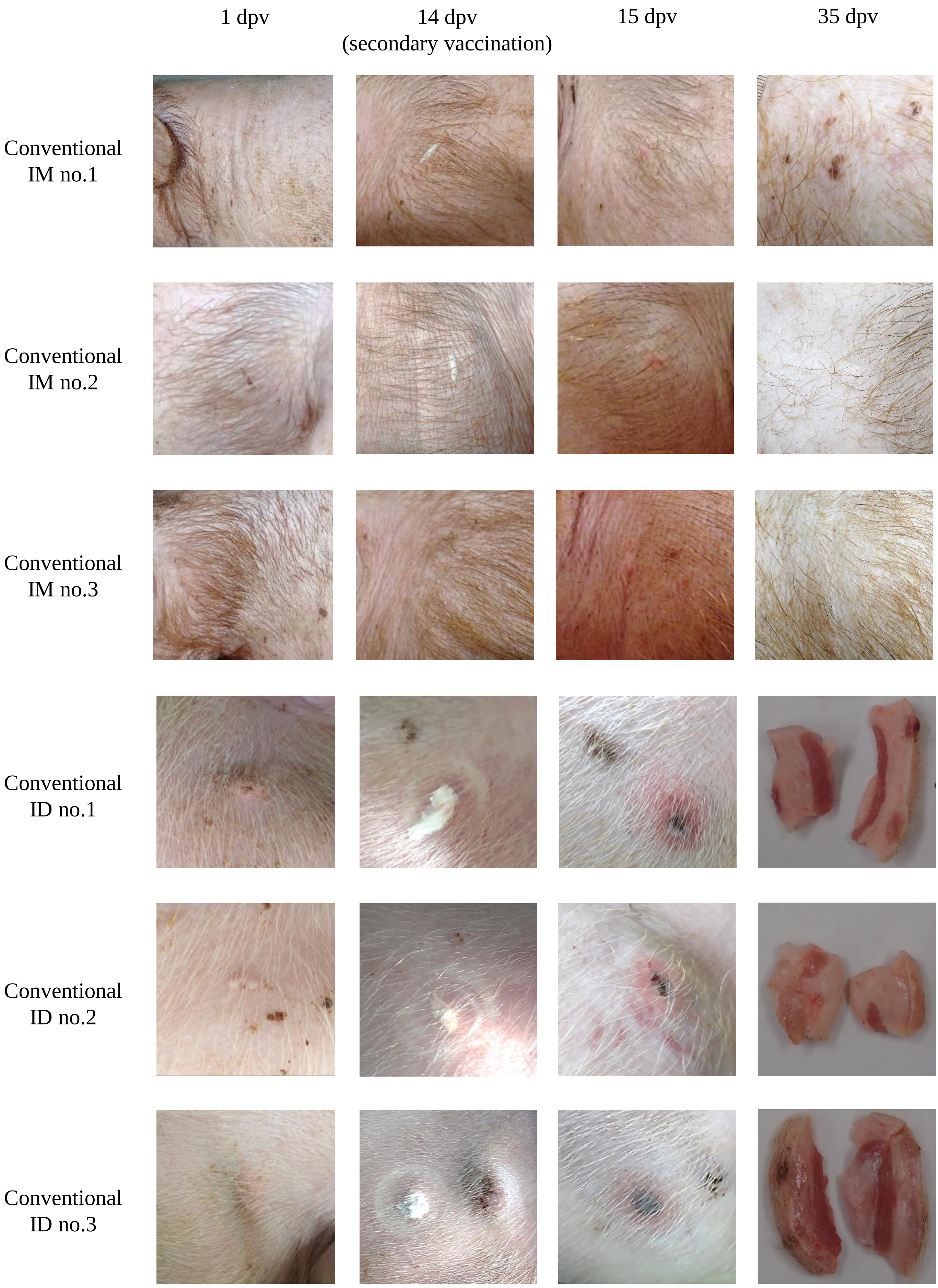
1 dpv
14 dpv (secondary vaccination)
15 dpv
35 dpv
Conventional IM no.1
Conventional IM no.2
Conventional IM no.3
Conventional ID no.1
Conventional ID no.2
Conventional ID no.3
