# Supplementary figures and images for: Intradermal Inoculation of Inactivated Foot-and-Mouth Disease Vaccine Induced Effective Immune Responses Comparable to Conventional Intramuscular Injection in Pigs
Source: Vaccines (Basel). 2024 Feb 13;12(2):190. doi: 10.3390/vaccines12020190 (PMC10892606; doi:10.3390/vaccines12020190)

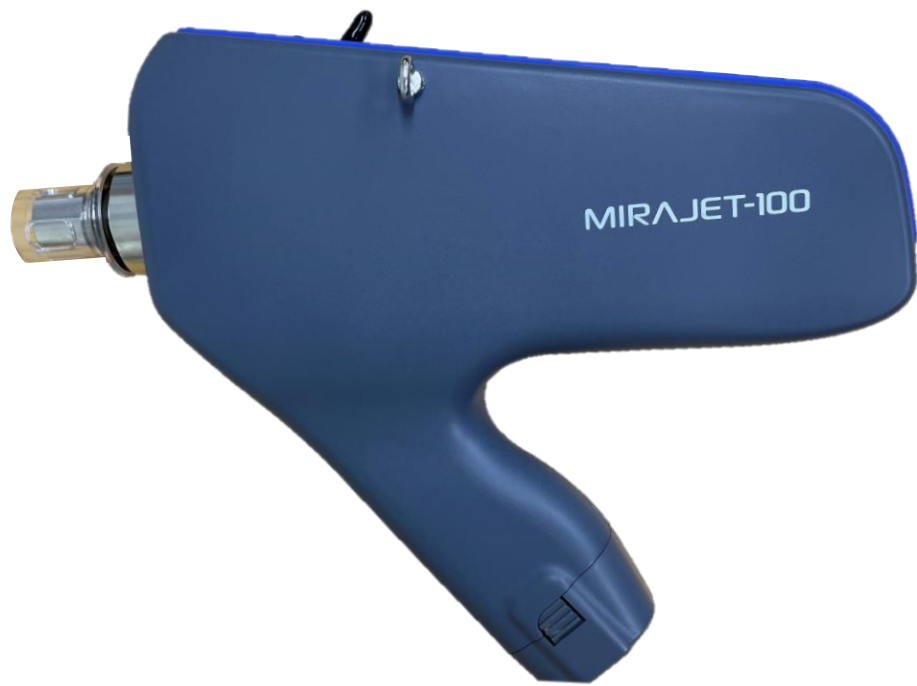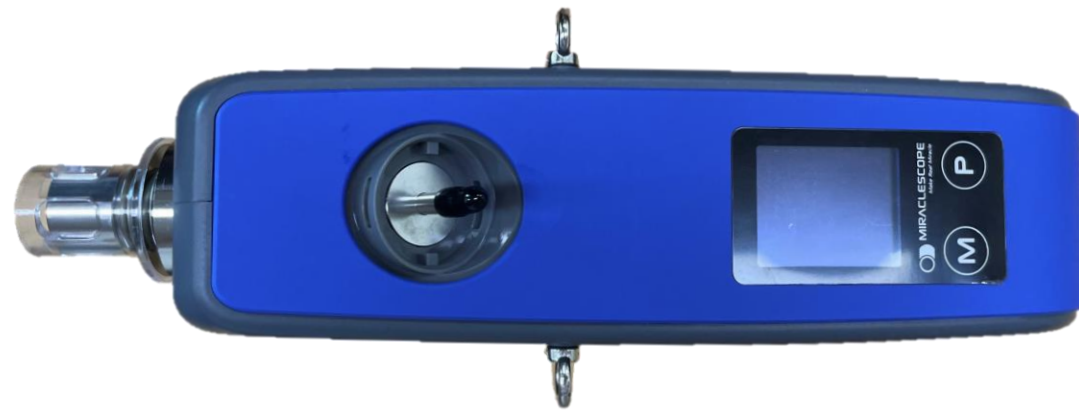

Supplement: Supplementary file 1 [file vaccines-12-00190-s001.zip › vaccines-2789294-supplementary Figure S1.pdf]

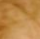

Supplement: Supplementary file 1 [file vaccines-12-00190-s001.zip › vaccines-2789294-supplementary Figure S2.pdf]

Conventional  
pigs

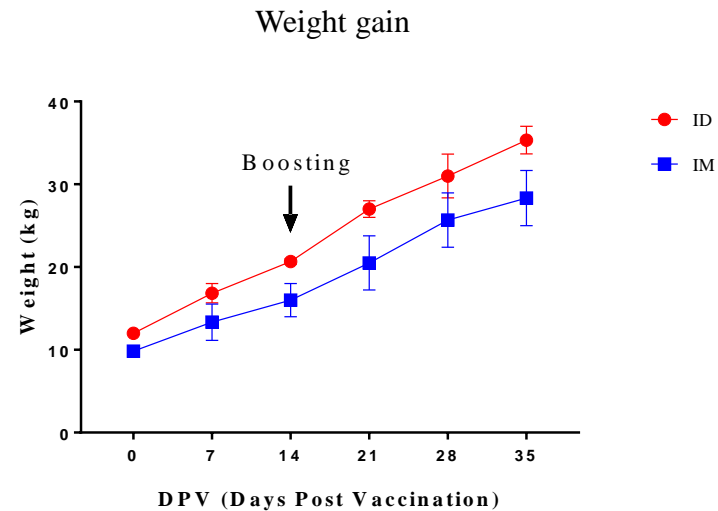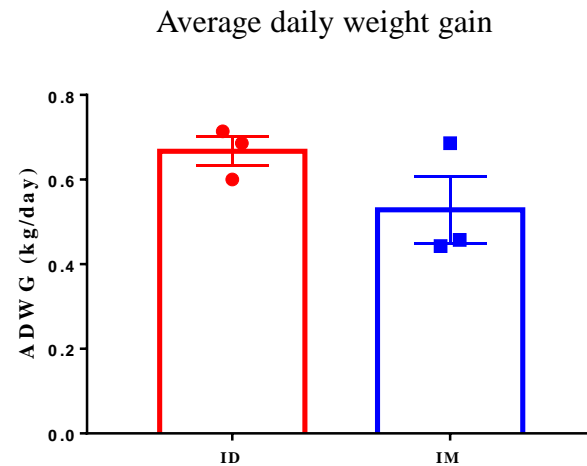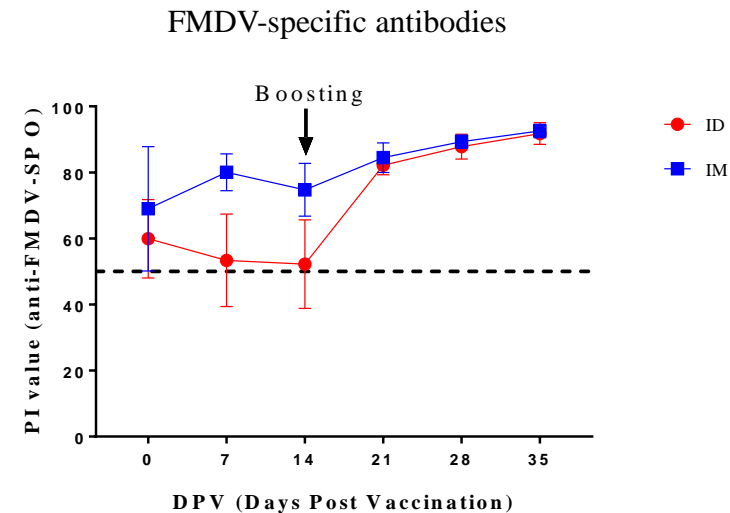

Supplement: Supplementary file 1 [file vaccines-12-00190-s001.zip › vaccines-2789294-supplementary Figure S4.pdf]
